# Musculoskeletal disorders and its associated factors among hospital cleaners in Addis Ababa, Ethiopia

**DOI:** 10.1038/s41598-024-53531-0

**Published:** 2024-02-05

**Authors:** Abel Afework, Aiggan Tamene, Abera Tafa

**Affiliations:** 1 Center for Sustainable Development, Addis Abeba University, Addis Abeba, Ethiopia; 2 Centre for Sustainability, University of Otago, Dunedin, New Zealand; 3 Department of Preventive and Social Medicine, University of Otago, Dunedin, New Zealand; 4 Infection Prevention and Control, Dilla University, Dilla, Ethiopia

**Keywords:** Health care, Health occupations

## Abstract

There is a paucity of published evidence about musculoskeletal disorders among hospital cleaners in Ethiopia. Therefore, this study was conducted to assess the prevalence of musculoskeletal disorders and its associated factors among hospital cleaners in Addis Ababa, Ethiopia. A total of 437 hospital cleaners participated in the study. A standardized questionnaire adapted from the Nordic musculoskeletal questionnaire was used for data collection. Bivariate and multivariable logistic regression analyses were used to determine factors associated with musculoskeletal disorders. The prevalence of work-related musculoskeletal disorders among hospital cleaners was 57.2% with 95% CI (52.6–62.0). Occupational safety training [AOR: 2.34, 95% CI (1.47–3.73)], repetitive tasks [AOR: 3.09, 95% CI (1.61–5.94)], heavy lifting [AOR: 5.21, 95% CI (3.20–8.48)], work-related stress [AOR: 2.42, 95% CI (1.48–3.97) and work-related dissatisfaction [AOR: 1.97, 95% CI (1.23–3.13)] were identified as associated factors for the development of musculoskeletal disorders. In conclusion the study revealed a high prevalence of musculoskeletal disorder. Notably, work related and organizational factors emerged as key contributing factors to the development of disorders. The identified associations underscore the importance of targeted interventions promoting organizational change involving managers to mitigate the risk of musculoskeletal disorders and enhance overall occupational health and well-being.

## Introduction

Musculoskeletal Disorders (MSDs) are health problems of the muscles, tendons, skeleton, cartilage, ligaments, and nerves including all forms of illness ranging from light to disabling injuries^1^. Globally, the large portion of MSDs is related to job exposure^2^. At the individual level, long-term sick leaves, and early retirements are frequently triggered by Work-related Musculoskeletal Disorders (WMSDs)^3^.

International Labour Organization reported that the annual estimated work related fatalities and non-fatal injuries are 2.78 million and 374 million, respectively. Furthermore, the estimated global economic loss linked to workplace injuries was roughly 3.9% of global GDP, with 1473 billion Euro and 1207 billion Euro lost owing to fatal and non-fatal incidents, respectively^4,5^.

Work-related Musculoskeletal conditions are identified as one of the leading causes of workplace complaints and lower back pain was reported to be the fourth leading cause of disability adjusted life year in 2019^6,7^. In the developing world, inadequacy in the understanding of occupational hazards by employees, lack of control measures regarding occupational hazards, and poorly designed workstations are likely to increase the burden of these disorders^8^.

Hospital cleaners help avoid the spread of communicable diseases in healthcare institutions through different cleaning procedures. The hazardous nature of the work performed during cleaning exposes them to a range of hazards including physical, mechanical, chemical, biological, ergonomic, and psychosocial hazards that favor the development of certain diseases such as WMSDs^9–12^.

Despite their significant importance, there is little consideration for the safety and health of hospital cleaners in many healthcare facilities^10^. Studies conducted in different regions showed a high prevalence of WMSD among hospital cleaners ranging from 51 to 81.9%^11,13,14^. Similarly a study conducted in Ethiopia showed a prevalence of 53.2%, and different factors like time pressure, work experience, working hour and positions were significantly associated with the development of WMSD^15^.

The Coronavirus pandemic has placed tremendous pressure on healthcare systems due to the increased patient flow and high work-demanding medical procedures. These refers to additional burdens on all available healthcare workforces, which might expose them to different injuries including WMSDs^16^. Despite numerous inquiries on WMSDs among hospital workers, however, previous studies concentrated on certain occupational groups such as doctors, nurses, physical therapists, dentists, and orderlies^17–19^. There is a paucity of published evidence about WMSD among hospital cleaners in Ethiopia. Therefore this study aimed to assess the prevalence of work-related musculoskeletal disorders and associated factors among hospital cleaners of tertiary hospitals in Addis Ababa, Ethiopia, 2021.

## Materials and methods

### The aim, study design, and setting

The aim of this study was to assess the magnitude of WMSDs and associated factors among hospital cleaners in Addis Ababa, the Ethiopian capital. An Institution-based cross-sectional study was conducted from August 25 to September 20, 2021. There are currently 4 Tertiary Hospitals in Addis Ababa. These hospitals were selected purposively as a result of their large patient flow and the high demand for efficiency prevalent in their cleaning divisions.

### Source and study population

All hospital cleaning staffs employed in tertiary hospitals of the capital city were the study population.

**Inclusion criteria** Workers with 12 months of minimum service were included.

**Exclusion criteria** Cleaners with spinal deformities or injuries that affect the musculoskeletal system were excluded.

### Sample size determination

Since no studies on WMSDs among hospital cleaners were found in our context, any effort to take the baseline prevalence from another setting may affect the representativeness of the present study. Therefore, the maximum sample size assumption, with prevalence, of the study outcome at p = 50%, a margin of error (d) of 5%, and a 95% confidence level were considered. Based on these assumptions, the EPI Info version 7 yielded a sample size of 384. After considering possible non-response during the period of data collection, a 10% non-response rate was applied to the final sample size. In the end, the final sample size calculated was 422.

### Sampling procedure

An on-site census was conducted to assess the study participants’ eligibility. A total of 468 cleaners were identified as working as housekeepers in the selected hospitals. Based on eligibility requirements, there were 31 hospital cleaners removed from consideration. That meant there were 437 hospital cleaners eligible for selection. The final study included all available 437 cleaners.

### Data collection tool and procedures

The study team consisted four health and safety specialist data collectors and one qualified field supervisor. Data was collected using a pre-tested and close-ended interviewer administered questionnaire via face-to-face interviews. The Standardized Nordic Musculoskeletal Questionnaire-Extended (NMQ-E) was used to assess work-related musculoskeletal symptoms^20^. The respondents were aided by an anatomical figure of nine body segments (neck, shoulder, upper and lower back, hands/wrists, arms, knee, thighs, and feet) to reliably describe the occurrence of musculoskeletal symptoms in the previous 12 months, one month and seven days.

The study participants' socio-demographic and personal characteristics were obtained using a questionnaire adapted from prior MSD investigations^17–19^. The general Job satisfaction scale together with the workplace stress scale, was used to assess job satisfaction and stress^21,22^. Anthropometric measures were taken using a digital weight scale and a standard meter to examine weight and height, respectively.

### Operational definition

**Work-related musculoskeletal disorders (WMSD)** are a self-reported pain in any part of the neck, shoulder, upper back, lower back, hip/thigh, knee/leg, and ankle/foot and wrist/hand for at least 2–3 workdays in the past week, month or year. These symptoms appear during work activity and, during rest, often disappear. After work ends, they may continue. They do not involve disabilities caused by slides, falls, motor vehicle crashes, or similar events^17^.

### Data quality control

Data quality was ensured in different ways. First, a health professional familiar with the medical terminologies made the forward translation of the Extended Nordic musculoskeletal questionnaire from English to Amharic. Instead of literal equivalence of the terminologies, the approach in the translations emphasized cross-cultural translations; subsequently, reverse translation into English was done by an expert in the English language.

Besides, 2 days of training on data collection instruments and data collection techniques were given to the supervisor and data collectors. The questionnaire was then pre-tested on 5% of the total sample size outside the study area in Bishoftu town hospital. The primary aim of the pre-test was to find any concerns with the tool’s design and readability. A secondary purpose of the pre-test was to ensure that people with or without anatomical expertise were able to interpret the instrument. The tool was finalized after the pre-test and necessary modifications. In the final analysis, data from the pre-test was not included.

### Data management and analysis

The collected data was checked, edited, coded, and entered into Epi-info version 7 and exported to Statistical Package for Social Science (SPSS 20) for additional analysis. WMSD data was obtained for the preceding 12 months, one month, and seven days. Nevertheless, only the annual prevalence was further investigated. Annual prevalence was chosen in this study because it was a suitable time scale similarly practiced in previous works.

Bivariate binary logistic regression analysis was done and variables with a p-value < 0.25 were exported to the multivariable binary logistic regression. At 95 percent CI and p-value 0.05, the significance level was obtained. To assess the strength of association, the adjusted odds ratio was used.

### Ethical consideration

The study complies with the Declaration of Helsinki Ethical Principles for Medical Research involving human subject. Ethical clearance and approval letter was obtained from Wachemo University College of Medicine and Health Sciences’ institutional review board (IRB). A letter of permission had been obtained from the Addis Ababa City Health Bureau before data collection. Also, full informed consent was obtained from respondent before every interview.

## Results

### Descriptive statistics of the study participants

#### Socio-demographic characteristics

The response rate of the study was 100%. Of the 437 study partakers, 433 (99.1%) were females. The mean age of the study participants was 31.9 ± 8.9. Regarding participants education, 119 (34.6%) of respondents completed their secondary education, while 133 (30.4%) did not obtain any formal education. A majority, that is, 151 (34.6%) had worked between 5 and 15 years. It was also noted that 312 (71.4%) of the respondents were permanently employed while 125 (28.6%) were contract (Table 1).

**Table 1:** Socio-demographic characteristics of hospital housekeepers in Addis Ababa, Ethiopia, September 2021.

Categories for variables	Frequency (n = 437)	Percent (%)
Sex		
Female	433	99.1
Male	4	0.9
Age in completed years		
< 20	16	3.8
21–25	88	20.1
26–30	144	33.0
31–35	111	25.4
36–40	54	12.3
≥ 41	24	5.4
Educational status		
No formal education	133	30.4
Elementary education	149	34.1
Secondary education	144	33.0
Tertiary and above	11	2.5
Service year		
< 5 years	135	30.8
5–10 years	151	34.6
≥ 11 years	151	34.6
Monthly net income (in dollar)		
< 400	123	28.1
400–500	242	55.4
501–600	46	10.5
> 600	26	5.9
BMI		
Underweight (BMI < 18.50)	28	6.4
Normal weight (BMI 18.50–24.99)	339	77.6
Overweight (BMI 25.00–29.99)	59	13.5
Obese (BMI ≥ 30.00)	11	2.5
Employment type		
Permanent	312	71.4
Contract	125	28.6

#### Occupation-related characteristics of respondents

From the total participant, 191 (43.7%) had taken training on occupational health and safety. Respondents were asked about the number of hours they spent standing at the worksite every day and 230 (52.6%) affirmed standing between 1 and 3 h per day. It was also found that 314 (71.9%) of the respondents worked in the day shift, while the remainder were night shifters and 274 (62.7%) spent greater than 8 h at their work sites. Regarding stress, 168 (38.4%) of the study respondents suffered from job stress (Table 2).

**Table 2:** Occupation related characteristics of hospital housekeepers in Addis Ababa, Ethiopia, September 2021.

Categories of variables	Frequency (n = 437)	Percent (%)
Occupational safety and health training		
Yes	191	43.7
No	246	56.7
Work Shift		
Day shift	314	71.9
Night shift	123	28.1
Hours spent Standing at work/day		
1–3 h	230	52.6
4–6 h	182	41.6
> 6 h	25	5.7
Number of days spent at work		
< 5 days	133	30.4
6 days	264	60.4
7 days	40	9.2
Hours spent at work per day		
< 8 h	163	37.3
> 8 h	274	62.7
Shortage of staff in the unit assigned		
Yes	217	49.7
No	220	50.3
Stress-related to Job		
Yes	168	38.4
No	269	61.6
Satisfaction stemming Job		
Yes	217	49.7
No	220	50.3

#### Ergonomics related characteristics of respondents

Of the study participants, 202 (46.2%) of the cleaners' jobs often involved bending and turning in uncomfortable manner, while 212 (48.5%) of the respondents worked for more than 2 h a day in the same position. With regards to heavy loads, 293 (67.0%) of the respondents regularly pushed, pulled, raised, and shifted loads greater than 20 kg (kg) without any assistance or the use of assistive equipment.

Participant who reported lifting loads of exceeding 5 kg (kg) were 205 (46.9%). Similarly, 210 (48.1%) of the participants regularly used vibrating materials when conducting their work in the hospitals and 262 (60.0%) of the participants reported using assistive equipment while at work. Concerning work postures, 135 (30.9%) of the respondents reported that standing was their most frequently adopted work pose on the worksite while kneeling, bending, and squatting accounted for 14.0%, 23.8%, 6.9% respectively (Table 3).

**Table 3:** Ergonomics related characteristics of hospital housekeepers in Addis Ababa, Ethiopia, September 2021.

Categories of variables	Frequency (n = 437)	Percent (%)
Most commonly adopted work posture		
Standing	135	30.9
Sitting	33	7.6
Kneeling	61	14.0
Bending	104	23.8
Squatting	30	6.9
Crawling	74	16.9
Repetitive movements		
Never	95	21.7
Sometimes	175	40.0
Always	167	38.2
Lift, push, pull, carry loads > 5 kg		
Yes	293	67.0
No	144	33.0
Lift, push, pull, carry loads > 20 kg		
Yes	205	46.9
No	232	53.1
Bending/twisting in an awkward way		
Never	74	16.9
Sometimes	161	36.8
Always	202	46.2
Work in the same position for > 2 h		
Never	73	16.7
Sometimes	154	35.2
Always	210	48.1

### Prevalence of self-reported work-related musculoskeletal disorders

The period prevalence of hospital cleaners who had experienced pain in at least one part of their body over the 12 months before the study was 250 (57.2%) with 95 CI (52.6, 62.0), whereas the 30 days and 7 days point prevalence were 202 (46.2%) and 52 (11.9%), respectively.

Of the self-reported 12 months work-related musculoskeletal pain or discomfort, the three most prevailing complaints were shoulder pain (29.1%), followed by lower back (28.6%) and wrist/hand complaints (17.2%) (Table 4).

**Table 4:** Prevalence of work related musculoskeletal disorders in different body segments of hospital house keepers in Addis Ababa, Ethiopia, September 2021 (n = 250).

Body regions	Frequency	Prevalence [CI 95%]
Neck	26	5.9% [3.9–8.5]
Shoulder	127	29.1% [24.7–33.4]
Upper back	17	3.9% [2.1–5.7]
Elbow	18	4.1% [2.3–5.9]
Lower back	125	28.6% [24.7–32.7]
Wrist/hands	75	17.2% [13.7–20.8]
Hips/thighs/buttocks	36	8.2% [5.7–11.03]
Knees	63	14.4% [11.0–17.8]
Ankles/feet	52	11.7% [8.9–14.9]

On the subject of the reported number of disorders, 76 (16.2%) of the cleaners had WMSDs in one body part, 67 (14.4%) in two, 54 (11.4%) in three, 38 (7.8%) in four, and 15 (2.9%) in five out of the nine body parts investigated.

### Factors associated with work-related MSDs among Hospital Housekeepers

Several factors resulted in significant associations with p-values of < 0.25 during bivariate analysis and entered to the multivariable analysis. Multivariable binary logistic regression identified occupational safety and health training, handling loads exceeding 20 kg, repetitive motions, work-related stress, and job-related dissatisfaction, as having significant associations with WMSDs (Table 5).

**Table 5:** Multivariable logistic regression analysis of the adjusted effect of factors associated with work-related musculoskeletal disorders among hospital housekeepers in Addis Ababa, Ethiopia, September 2021.

Variable	Work-related MSD		COR (95% CI)	AOR (95% CI)	P-value
	Yes	No			
Occupational safety and health training					
Yes	90 (47.1%)	101 (52.9%)	2.0 (1.41–3.07)	**2.34 (1.47–3.73)**	**0.0001**
No	160 (65.0%)	86 (35.0%)	1	1	
Hours spent at work per day					
< 8 h	83 (50.9%)	80 (49.1%)	1	1	
> 8 h	167 (60.9%)	107 (39.1%)	1.5 (1.01–2.22)	1.17 (0.72–1.89)	0.521
Repetitive motions					
Never	43 (45.3%)	52(54.7%)	1	1	
Some times	101 (57.7%)	74 (42.3%)	1.65 (0.99–2.73)	**3.09 (1.61–5.94)**	**0.001**
Always	106 (63.5%)	61 (36.5%)	2.10 (1.25–2.50)	**2.78 (1.46–5.29)**	**0.002**
Availability of assistive equipment					
Yes	137 (52.3%)	125 (47.7%)	1	1	
No	113 (64.6%)	62 (35.4%)	1.66 (1.12–2.46)	1.18 (0.71–1.96)	0.511
Regularly lift, push, pull loads > 5 kg					
Yes	158 (53.9%)	135 (46.1%)	0.66 (0.49–0.99)	0.71 (0.42–1.19)	0.20
No	92(63.9%)	52(36.1%)	1	1	
Regularly lift, push, pull loads > 20 kg					
Yes	158 (77.1%)	47 (22.9%)	5.11 (3.36–7.77)	**5.21 (3.20–8.48)**	**0.0001**
No	92 (39.7%)	140( 60.3%)	1	1	
Shortage of staff in the assigned unit					
Yes	139 (64.1%)	78 (35.9%)	1.75 (1.19–2.56)	1.03 (0.64–1.67)	0.887
No	111 (50.5%)	109 (49.5%)	1	1	
Satisfaction stemming from job					
Yes	109 (50.2%)	108 (49.8%)	1	1	
No	141 (64.1%)	79 (35.9%)	1.76 (1.20–2.59)	**1.97 (1.23–3.13)**	**0.004**
Stress-related to work					
Yes	118 (70.2%)	50 (29.8%)	0.48 (0.27–0.61)	**2.42 (1.48–3.97)**	**0.0001**
No	132 (49.1%)	137 (50.9%)	1	1	

Significant values are in bold.

Hospital cleaners who did not have training in occupational health and safety were 2.3 times more likely to have MSD grievances than their colleagues who took part in such training [AOR: 2.34, 95% CI (1.47–3.73)]. Similarly, cleaners whose tasks regularly involved repetitive movements were 3.09 times more likely to develop WMSD grievances than those whose tasks did not include repetitive movements [AOR: 3.09, 95% CI (1.61–5.94)].

In the same way, those participants who sometimes involved in repetitive movements were 2.78 times more likely to develop WMSD complaints [AOR: 2.78, 95% confidence CI (1.46–5.29)] compared to their colleagues who never involved in such activities. The likelihood of WMSD pain among cleaners who regularly lifted, moved and/or pulled loads greater than 20 kg without the support of another person or assistive equipment was 5.21 times that of those who did not participate in such activities regularly [AOR: 5.21, 95% CI (3.20–8.48)].

The odds of experiencing MSD pain in the last 12 months was 2.4 times higher in cleaners who were stressed as a result of their job and 1.97 times in cleaners who were dissatisfied with their job [AOR: 2.42, 95% CI (1.48–3.97) and 1.97 (1.23–3.13)], respectively.

## Discussion

In this study, the overall prevalence of WMSDs was 57.2% with 95 CI [(52.6, 62.0)]. The finding is slightly higher compared to a study conducted in Mekelle Ethiopia which reported annual prevalence of 52.3%^15^. The annual prevalence of WMSDs obtained in this study was lower than the prevalence reported 68.3%, 77.4% and 81.9% in India^13^, Brazil^23^, and Thailand^14^. The variations in epidemiological case descriptions that may occur between the various studies should be taken into consideration when contrasting this result with the results of other musculoskeletal epidemiologic surveys. Epidemiological case description differences have a substantial effect on the prevalence of common musculoskeletal disorders^24^.

The top 5 body parts reported by respondents complaining ailments were shoulders, lower back, wrist, knee and hip. This finding is similar to a finding from an Indian study in which high number of complaints were reported about aliments of the same body parts^11^. This might be due to the reason that the physical demands of their job, which involve repetitive movements, heavy lifting, and uncomfortable postures put stress on the joints and tissues in the shoulder, lower back, wrist, knee, and hip areas, leading to pain and discomfort by most cleaners^25^.

The most-reported pain or discomfort in this study was shoulder pain at 29.1%. This was consistent with a study done in India^13^. In a previous study of musculoskeletal risk factors in cleaning occupation the researchers discovered that one or both arms were above shoulder level in 24–43% of the time in cleaning work^25^. Thus, it seems logical to think that shoulder ailments may be caused or exasperated by the work environment in Hospitals.

Back pain was recorded as the second most problematic condition at 28.6%. The possible reason for this could be when cleaning hospitals; housekeepers regularly have to assume non-neutral trunk postures. Previous research has reported that fixtures, furnishings, and other design elements within hospitals often require cleaners to adopt gruelling work postures that can result in pain and discomfort^25^.

Employees who have obtained specialized training are more likely to comply with the recommended safety rules and to have a greater knowledge of work-related accidents and disorder prevention^26^. Likewise, in this study indicated that not having occupational safety and health training was significantly related to musculoskeletal disorders. Hospital housekeepers who did not have occupational safety and health training were 2.34 times more likely to develop musculoskeletal disorders than their colleagues who took part in such training (Supplementary information).

Another important determinant of WMSDs in the present study was repetitive motions. Cleaners whose tasks regularly involved repetitive movements were 3.09 times more likely to develop WMSD grievances than those whose tasks did not include repetitive movements. In the same way, relative to their colleagues who never participated in such activities, those who sometimes participated in repetitive movements were 2.78 times more likely to develop WMSD complaints^27^. A repetitive motion has been described previously as one of the major contributing factor to musculoskeletal disorders^28^.

With regards to heavy manual material handling, the odds of musculoskeletal disorders among housekeepers who engaged in moving loads that exceed 20 kg without people’s help or assistive tools was 5.21 times higher than those who did not engage in such activities. This is supported by findings from Brazil and Canada^23,25^. Studies affirmed that hospital cleaners are responsible for the manual transport of loads in many parts of the developing world. The amount of load decides whether tissues bear a load that is beyond their capacity or not. If the ability of the tissue is not surpassed, a stable adaptive state is retained. However when it is reached, a temporary or permanent unhealthy condition takes place^13,29^.

According to studies, occupational stress occurs when job expectations do not match the worker's skills, capabilities, or needs, and it causes emotional disturbances, behavioral issues, biochemical and neuro-hormonal changes that may increase the risk of physical disease^30–32^. In the current study, hospital cleaners who were stressed by their job were 2.4 times more likely to suffer musculoskeletal problems at work than those who were not stressed out by their job. This is confirmed by findings from two Indian studies in which psychosocial factors were found to be significantly associated with all types of MSDs^11,13^.

In the present study a positive association between job dissatisfaction and musculoskeletal symptoms was observed. Cleaners who were dissatisfied with their job were 1.97 times more likely to have MSD complaints than their colleagues with no such grievance. A study affirmed that those cleaners who were more satisfied with their jobs are most likely to adhere to safety precaution and are less likely to experience work-related accidents and injuries^33^.

## Conclusions

The study revealed a high prevalence of musculoskeletal disorder. Notably, work-related and organizational characteristics such as training, repetitive tasks, stress, job satisfaction, and heavy lifting revealed as important contributors to the development of WRMD. The identified associations highlight the importance of targeted interventions that promote organizational change by involving employees, managers, and stakeholders in collaborative efforts to create a healthy work environment in order to reduce the risk of musculoskeletal disorders and improve overall occupational health and well-being.

### Supplementary Information

Supplementary Information.

## Data Availability

The data sets used and/or analyzed in the present study can be obtained from the corresponding author upon reasonable request (agziabel@gmail.com).

## Abbreviations

BMI: Body mass index
AOR: Adjusted odds ratio
ILO: International Labor Organization
MSD: Musculoskeletal disorders
WMSDs: Work-related musculoskeletal disorders
ICUs: Intensive care units
COVID-19: Corona virus 2019
NMQ-E: The extended version of the Standardized Nordic Musculoskeletal Questionnaire
SPSS 20: Statistical Package for Social Science
U.S: United States of America
