# Supplementary material for: Musculoskeletal disorders and its associated factors among hospital cleaners in Addis Ababa, Ethiopia
Source: Sci Rep. 2024 Feb 5;14:2887. doi: 10.1038/s41598-024-53531-0 (PMC10838922; doi:10.1038/s41598-024-53531-0)
Supplement: Supplementary file 1 — Supplementary Information. [file 41598_2024_53531_MOESM1_ESM.docx]

# English version of the questionnaire

**Date of interview _________________**

**Interviewer’s name _______________**

**Questioner number­­­­­­ _______________**

**Section One: - Socio-demographic characteristics of respondents**

| **No** | **Questions** | | **Responses** | | **Skip** |
| --- | --- | --- | --- | --- | --- |
| **101** | Sex | | 1.Male  2.Female | |  |
| **102** | Age in completed years (NB. Please round up if the month is Known) | | _________________ | |  |
| **103** | Educational status | 1. Un able to read and write 2. Read and write only 3. Elementary (grade 1-8) | 1. Secondary (grade 9-12) 2. College/ TVET | 1. Degree and above 2. Other ________ |  |
| **104** | Monthly Net Income? | | -------------Ethiopian Birr | |  |
| **105** | Work Experience | | -------------- Years --------------- Months | |  |
| **106** | Work Title | | 1. Senior Housekeeper 2. Assistant/ Apprentice/Helper | |  |

**Section two: Personal factors associated with work-related musculoskeletal disorders**

| **No** | **Questions** | **Responses** | **Skip** |
| --- | --- | --- | --- |
| **201** | Weight in Kg | ------------kg |  |
| **202** | Height? | ---------M ---------cm |  |
| **203** | Do you smoke cigarette?  At least one stick of cigarette a day | 1. Yes 2. No |  |
| **204** | Do you drink alcohol?  at least twice a week | 1. Yes 2. No |  |
| **205** | Do you practice physical exercise? at least twice a week for 30 minutes | 1. Yes 2. No |  |
| **206** | Have you ever chewed khat? | 1. Yes 2. No | 208 |
| **207** | If yes, have you chewed Khat in the last 30days? | 1. Yes 2. No |  |
| **208** | Have you ever taken training in occupational health and safety? | 1. Yes 2. No |  |
| **209** | Past medical history of systemic illness?  (*Diabetes, low thyroid or overactive thyroid, chronic renal failure, gout and dyspepsia*) | 1. Yes 2. No |  |

**Section three –Work environment (Occupational) factors associated with work-related MSD**

| **No** | **Questions** | **Response** | | **Skip** |
| --- | --- | --- | --- | --- |
| **301** | How many days do you spend on work per week? | | -----------Days |  |
| **302** | How many hours do you spend on your work per day? | | -----------Hours |  |
| **303** | How much time a day do you spend standing in you work? | | --------Hour |  |

**Section Four- Ergonomic factors associated with work-related musculoskeletal disorders**

| **No** | **Questions** | **Response** | | **Skip** |
| --- | --- | --- | --- | --- |
| **401** | Does your job involve you bending or twisting in an awkward way? | 1. Never 2. Sometimes 3. Always | |  |
| **402** | Do you work in the same position for more than 2 hours (standing, bent over, sitting, squatting and kneeling) | 1. Never 2. Sometimes 3. Always | |  |
| **403** | What is your most commonly adopted posture? | 1. Standing 2. Sitting 3. Kneeling 4. Bending | 1. Squatting 2. Reaching overhead |  |
| **404** | Does your job require you repeating motions with less than 30 seconds difference? | 1. Never 2. Sometimes 3. Always | |  |
| **405** | Do you often, in your work, lift, push, pull, carry or move heavy loads (more than 5kg) without peoples help or an assistive tool? | 1. Yes 2. No | |  |
| **406** | Do you often, in your work, lift, push, pull carry or move very heavy loads (exceeding 20kgs) without peoples help or an assistive tool? | 1. Yes      1. No | |  |
| **407** | Do you have assistive tools/ machinery that make your work easier? | 1. Yes 2. No | |  |
| **408** | Do you in your work have: Insufficient space to do your work properly? | 1. Yes 2. No | |  |

***Section five* Psychosocial factors associated with work-related musculoskeletal disorders**

| **No.** | **Questions /variables** | **Job stress score** | | | | |
| --- | --- | --- | --- | --- | --- | --- |
|  |  | Never | Rarely | Sometimes | Often | Very often |
| **409** | Conditions at work are unpleasant or  sometimes even unsafe | 1 | 2 | 3 | 4 | 5 |
| **410** | I feel that my job is negatively affecting my physical or emotional wellbeing | 1 | 2 | 3 | 4 | 5 |
| **411** | I have too much work to do and/or too many  unreasonable deadlines | 1 | 2 | 3 | 4 | 5 |
| **412** | I find it difficult to express my opinion or feelings about my job conditions to my superiors. | 1 | 2 | 3 | 4 | 5 |
| **413** | I feel that job pressures interfere with my family or personal life. | 1 | 2 | 3 | 4 | 5 |
| **414** | I have adequate control or input over my work duties. | 5 | 4 | 3 | 2 | 1 |
| **415** | I receive appropriate recognition or rewards for good performance. | 5 | 4 | 3 | 2 | 1 |
| **416** | I am able to utilize my skills and talents to the fullest extent at work | 5 | 4 | 3 | 2 | 1 |
|  | Final Score |  | | | | |

Job satisfaction

| **No.** | **Questions /variables** | **Job satisfaction score** | | | | |
| --- | --- | --- | --- | --- | --- | --- |
|  |  | Very  Dissatisfied | Dissatisfied | Neutral | Satisfied | Very satisfied |
| **417** | I receive recognition for a job well done. | 1 | 2 | 3 | 4 | 5 |
| **418** | I feel close to the people at work | 1 | 2 | 3 | 4 | 5 |
| **419** | I feel good about working here | 1 | 2 | 3 | 4 | 5 |
| **420** | I feel secure about my job. | 1 | 2 | 3 | 4 | 5 |
| **421** | I believe this work is good for my physical health | 1 | 2 | 3 | 4 | 5 |
| **422** | My wages are good | 1 | 2 | 3 | 4 | 5 |
| **423** | All my talents and skills are used at work | 1 | 2 | 3 | 4 | 5 |
| **424** | I get along with my supervisors | 1 | 2 | 3 | 4 | 5 |
| **425** | I feel good about my job | 1 | 2 | 3 | 4 | 5 |
|  | Final Score |  | | | | |

**Section six: Questioners to asses WMSD in neck, shoulder, upper back, lower back, hip /thigh, knee/leg, ankle/foot and wrist /hand. The diagram below shows the approximate position of the body parts referred to in the questionnaire.**


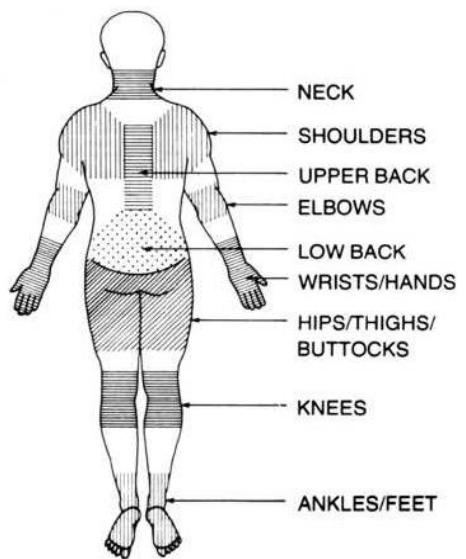


| Have you at any time in the last 12 months had trouble (ache, discomfort and pain ) lasting 2-3days that resulted in either:   - Being prevented from doing normal work (job, house work because of the trouble) - Hospitalization due to pain - Change of duties/ responsibilities within the job due to pain - An assessment by a health professional, physiotherapist, chiropractor or other such person - Taking Prescribed medication or sick leaves   In your: (Neck, Shoulder/s, Upper Back, Elbow/s, Wrist/s, Lower Back, Hip/s, Ankle/Feet)  N.B Considerations should not include disorders caused by slips, falls, motor vehicle accidents, or similar incidents. | | |
| --- | --- | --- |
| I. Neck | IV. Elbow/s | VII. Hip/Thigh/Buttock |
| 1. No 2. Yes | 1. No 2. In the Right Elbow 3. In the Left Elbow 4. In Both Elbows | 1. No 2. In the Right Hip/Thigh/Buttock 3. In the Left Hip/Thigh/Buttock 4. In Both Hips/Thighs/Buttocks |
| II. Shoulder/s | V. Wrist/Hands | VIII. Knee/s |
| 1. No 2. In the Right Shoulder 3. In the Left Shoulder 4. In Both Shoulders | 1. No 2. In the Right Wrist 3. In the Left Wrist 4. In Both Wrists | 1. No 2. In the Right Knee 3. In the Left Knee 4. In Both knees |
| III. Upper Back | VI. Lower Back | IX. Ankle/Feet |
| 1. No 2. Yes | 1. No 2. Yes | 1. No 2. In the Right Ankle/Feet 3. In the Left Ankle/Feet 4. In Both Ankles/Feet |

Section II

| Have you had trouble during *the last* ***30*** *days*  In your; | Have you had trouble during *the last* ***7*** *days* in your; |
| --- | --- |
| *Neck*   1. No 1. Yes | *Neck*   1. No 1. Yes |
| *Shoulder (Both/Either)*   1. No 1. Yes | *Shoulder (Both/Either)*   1. No 1.Yes |
| *Upper Back*   1. No 1. Yes | *Upper Back*   1. No 1. Yes |
| *Elbows(Both/Either)*   1. No 1. Yes | *Both/Either(Both/Either)*   1. No 1. Yes |
| *Wrist/Hands (Both/Either)*   1. No 1. Yes | *Wrist/Hands (Both/Either)*   1. No 1. Yes |
| *Lower Back*   1. No 1. Yes | *Lower Back*   1. No 1. Yes |
| *Hips/Thighs/Buttocks (Both/Either)*  0.No 1.Yes | *Hips/Thighs/Buttocks (Both/Either)*  0.No 1.Yes |
| *Knees (Both/Either)*  0.No 1.Yes | *Knees (Both/Either)*  0.No 1.Yes |
| *Ankle/Feet (Both/Either)*  0.No 1.Yes | *Ankle/Feet (Both/Either)*  0.No 1.Yes |
